# FRAILTY THERAPEUTICS: CLOSING THE PHYSICAL ACTIVITY GAP FOR FRAIL ADULTS

**DOI:** 10.1093/geroni/igae098.1277

**Published:** 2024-12-31

**Authors:** Megan Huisingh-Scheetz, Brittni Bryant, Brandon Foster, Corliss Taylor, Arianna Parkhideh, Benjamin Mendez, Sylvia Brown, Nikita Thomas

**Affiliations:** University of Chicago, Chicago, Illinois, United States; University of Chicago, Chicago, Illinois, United States; University of Chicago, Chicago, Illinois, United States; University of Chicago, Chicago, Illinois, United States; University of Chicago, Chicago, Illinois, United States; University of Illinois College of Medicine Peoria, Peoria, Illinois, United States; NORC at the University of Chicago, Chicago, Illinois, United States; University of Chicago, Chicago, Illinois, United States

## Abstract

Physical activity has been described as the ‘ideal’ intervention for frailty. In fact, exercise is the most effective frailty mitigation intervention studied to date. Yet, frail older adults face great challenges to physical activity over time. These challenges include decreased physiologic capability, greater barriers, fewer resources, narrower availability, reduced self-efficacy, poorer outcome expectations and restricted payor coverage for long-term activity programming. In this presentation, we will review the challenges frail adults face to participating in the most promising frailty intervention. To do this, we will summarize barriers noted in prior frailty exercise trials and will also report findings from our local survey study assessing physical activity behavior change factors among non-frail, pre-frail and frail older adults (n=101, 93% African-American). We will also review the current restricted payment structure for community-based long-term care services like exercise programs. We will then describe a novel exercise intervention designed to address several of these unique challenges, currently undergoing efficacy testing in a randomized controlled trial. The EngAGE trial is a multisite randomized controlled trial designed to compare an exercise intervention delivered over a voice-activated device in the home (EngAGE) versus paper-based exercises in n=124 frail, homebound, African-American older adults. We will share the EngAGE trial design, methods, measures and preliminary study fundings. We will close by summarizing key remaining issues that need to be addressed to close the physical activity gap for frail adults and will offer ideas for future research directions. Trial registration: ClinicalTrials.gov NCT05337514

